# Taming molecular flexibility to tackle rare diseases

**DOI:** 10.1016/j.biochi.2015.03.018

**Published:** 2015-06

**Authors:** Maria Vittoria Cubellis, Marc Baaden, Giuseppina Andreotti

**Affiliations:** a Dipartimento di Biologia, Università Federico II, 80126 Napoli, Italy; b Laboratoire de Biochimie Théorique, CNRS, UPR9080, Univ Paris Diderot, Sorbonne Paris Cité, 13 rue Pierre et Marie Curie, 75005 Paris, France; c Istituto di Chimica Biomolecolare-CNR, 80078 Pozzuoli, Italy

**Keywords:** Molecular dynamics, Disease, Diagnostic use, Protein stability, AGAL, lysosomal alpha-galactosidase, DGJ, 1-deoxy-galactonojirimycin, RMSD, Root mean square deviation, RMSF, root mean square fluctuation

## Abstract

Many mutations responsible of Fabry disease destabilize lysosomal alpha-galactosidase, but retain the enzymatic activity. These mutations are associated to a milder phenotype and are potentially curable with a pharmacological therapy either with chaperones or with drugs that modulate proteostasis. We demonstrate the effectiveness of molecular dynamics simulations to correlate the genotype to the severity of the disease. We studied the relation between protein flexibility and residual enzymatic activity of pathological missense mutants in the cell. We found that mutations occurring at flexible sites are likely to retain activity *in vivo*. The usefulness of molecular dynamics for diagnostic purposes is not limited to lysosomal galactosidase because destabilizing mutations are widely encountered in other proteins, too, and represent a large share of all the ones associated to human diseases.

## Highlights

• Residual alpha-galactosidase activity may relate to mild phenotype in Fabry disease.
• Molecular dynamics identifies flexible residues in lysosomal alpha-galactosidase.
• Mutations at flexible sites tend to maintain residual alpha-galactosidase activity.

## 1 Introduction

For most diseases different types of mutations exist and each type requires a specific therapeutic approach. Some mutations lower the free energy difference between the folded and the unfolded protein, shifting the equilibrium towards the latter one. Unstable proteins, although retaining the functional chemical groups needed for the biological activity, are sensitive to proteolysis and are cleared by the protein quality control systems in the cell. Hence, for these mutations, which represent a good share of all the ones associated with human diseases [1], the reduction of the protein concentration in the cell is the primary effect and the reduction of total activity is only a secondary effect. Small chemicals, which are known as pharmacological chaperones, bind preferentially to the folded state, thereby at least partially restoring the equilibrium between folded and unfolded states, and rescue these mutants [2]. They cannot be used for all the genotypes of a given disease, but in general are limited to those which retain residual activity. Nonetheless pharmacological chaperones offer advantages, low cost, oral administration and increased bio-availability. Beside pharmacological chaperones, other small molecules are being evaluated for therapy. They are not specific for a given mutated protein, but alter protein homeostasis [3].

Computational modeling, for instance molecular dynamics simulations, can be used to predict residual activity in the cell. This knowledge is important both for diagnosis and for therapy, because residual activity, the severity of the disease, and responsiveness to small molecule drugs are correlated.

Fabry disease represents a good example to show how conformational flexibility predictions can be used for designing original treatments for rare diseases. Among the many computational techniques that exist to predict protein flexibility such as normal mode analysis and distance geometry approaches, we will focus on molecular dynamics (MD). Fabry disease is X-linked and relatively frequent, 1–9 in 100000 (OMIM: 30150). Different mutations of the gene encoding lysosomal alpha-galactosidase A (AGAL) result in a wide phenotypic spectrum, with respect to age at onset, rate of disease progression, severity of clinical manifestations. Patients with the late onset or atypical form of Fabry disease retain some AGAL activity and are asymptomatic until adult age when they develop cardiac and kidney problems [4].

The treatment of Fabry disease with a pharmacological chaperone 1-deoxy-galactonojirimycin (DGJ) was first proposed by Fan et al., in 1999 [5]. The introduction in clinical practice of galactose to enhance AGAL activity in patients was reported by Frustaci et al., in 2001 [6]. Since then, responsiveness to pharmacological chaperones has been assessed for a huge number of AGAL mutations, covering both early and late onset forms of Fabry disease (for a review please consult Fabry_CEP [7] and references therein). A relatively large proportion of mutants, in particular among mutations associated with the late onset form of Fabry disease, recover activity when treated with DGJ. In a few cases it was possible to prove that DGJ acts by enhancing thermodynamic stability of the mutants [8,9].

In this paper we correlate the flexibility of the sites where AGAL mutations occur with the residual activity in the cells. This result is useful for the evaluation of severity and the choice of a personalized therapy. The direct measure of residual activity in the cells for each case would be impractical because more than 520 missense/nonsense mutations have been described in the databank HGMD^®^ professional [10] for Fabry disease and most of them are private, that means that they are seen in a single family.

## 2 Materials and methods

### 2.1 Molecular dynamics simulations

We used the structure of AGAL solved in the presence (3GXT) or in the absence (3GXN) of DGJ at pH 4.5 as input. We run a 50 ns MD simulation with the amber03 force field (a variant of the AMBER-99 one [11]) at the same pH at which crystals were grown. We used the Yasara program under default conditions combined with fully automatic optimized assignment of topology and parameters for the ligand using the AutoSMILES procedure [12,13]. All systems were solvated with explicit TIP3P water molecules and Na+ and Cl-counterions were added as background salt and to preserve overall electrical neutrality. Each system was energy minimized by using the steepest descent method to relax any steric conflicts before beginning the simulations. Simulations were carried out with periodic boundary conditions. Long-range electrostatic interactions were calculated by using PME with a direct-space cut-off of 7.86 Å. All simulations were performed by using an NVT ensemble at 298 K. A 2 fs/1 fs double-integration time step was used. Root mean square deviation (RMSD) between structures following least-squares fitting to the reference energy-minimized input structure, was calculated with Gromacs [14]. The RMSD value increases steadily in the first 25 ns. After the stabilization of the system, Root mean square fluctuations (RMSF) of alpha carbons of each residue were calculated with Gromacs [14].

### 2.2 Miscellaneous

Graph plotting was carried out with Kaleidagraph (Synergy Software, PA).

We assigned secondary structure with SEGNO [15]. Active site residues were identified with DrosteP [16]. The figure showing the AGAL structure colored by RMSF was produced with CHIMERA [17].

## 3 Results and discussion

### 3.1 Correlation between alpha-galactosidase flexibility and residual activity of missense mutants in the cell

An attempt to systematically classify the phenotype of Fabry disease based on structural features of AGAL was made by Saito [18]. In this previous work, mutations were divided into two categories defined as classic or atypical based on clinical signs. We believe that this classification is too simplistic because for most mutations few affected people are known and symptoms are variable even among patients with the same mutation [19]. In general, residual enzyme activity is associated with a less severe phenotype [20,21]. We decided to use a different approach and move from binary categories to real values, taking advantage of the fact that enzymatic activity of mutants has been measured in cells derived from patients or in cells transiently transfected with plasmid carrying a specific AGAL mutation.

We collected data from several laboratories [22–28] and examined 244 mutations altogether. The effect of mutations depends critically on the positions of the affected residue in the protein structure and secondarily on the type of substitution, conservative ones being more tolerated. In a few cases, lack of activity can be explained straightforwardly because the mutation affects the active site or disulphide bridges. We define active site residues as those that line the most conserved pocket in the protein structure: by this definition the active site residues in AGAL are W47, D92, D93, Y134, L168, D170, Y207, R227, E203, L206, D231, S297. When these amino acids are mutated, activity is null or proximal to zero. Disulphide bridges are essential to maintain the 3D-structure of the protein and it is not surprising that when a cysteine involved in a bridge (C52, C94; C56, C63; C142, C172; C202, C223; C378, C383) is mutated, fold, and consequently activity, is lost. It is worth observing that not all the cysteine residues in AGAL form disulphide bridges, the exception being C90 and C174.

When the active site is not severely compromised, the activity in the cells depends on two factors, the specific activity (U/mg) and the amount of the mature protein, which in turn depends on stability. Only in a very few cases it was possible to purify mutants and measure specific activity or stability independently, but in these cases it was shown that most missense mutations observed in Fabry patients do not affect the maximal velocity (k_cat_) or the affinity for substrate (K_M_) [29]. In order to analyze all mutations and try to correlate their effect in the cell with the position in the protein structure, we calculated an average residual activity per site. For example, we assign a value of 10.75% to Q327 because Q327K and Q327E have 0 or 21.5% residual activity, respectively. We divided the data in two sets. The first one includes 66 sites with zero average residual activity, the second one 92 sites with non null average residual activity. This distinction is motivated by the observation that the residual activity and stability in the cell correlates only for mutants that are not completely inactive.

**Fig. 1:**
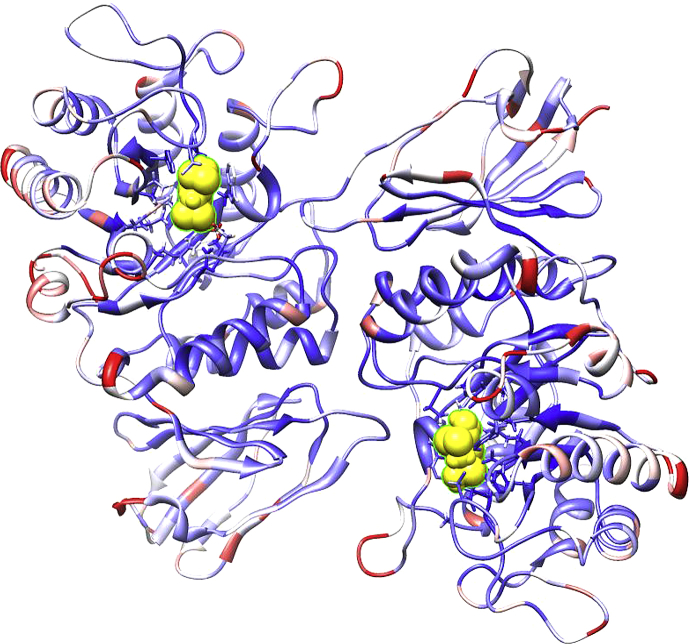
Crystal structure of AGAL (3GXT) in complex with DGJ (yellow). The structure was colored by RMSF ranging from blue (low flexibility) to white (medium flexibility) to red (high flexibility).

We carried out MD simulations using the structure of AGAL solved in the absence (3GXN) or in the presence (3GXT) of DGJ at pH 4.5 [30] as input. We run a 50 ns simulation at the same pH at which crystals were grown and calculated root mean square fluctuation (RMSF) values as a measure of flexibility per residue. In Fig. 1, the structure of AGAL solved in the presence of DGJ (3GXT) was colored by RMSF with colors ranging from blue (low flexibility) to white (medium flexibility) to red (high flexibility). AGAL is a homo-dimer and each subunit is made up by two domains, a TIM barrel where the active site is located and an antiparallel beta domain. Inspection of Fig. 1 suggests that flexibility is minimal in the regions not exposed to solvent buried between subunits, between domains or inside the TIM barrel.

Indeed accessibility (percent of residue surface exposed to solvent) and flexibility follow the same trend but direct correlation between the two properties is relatively low, approximately 70%.

**Fig. 2:**
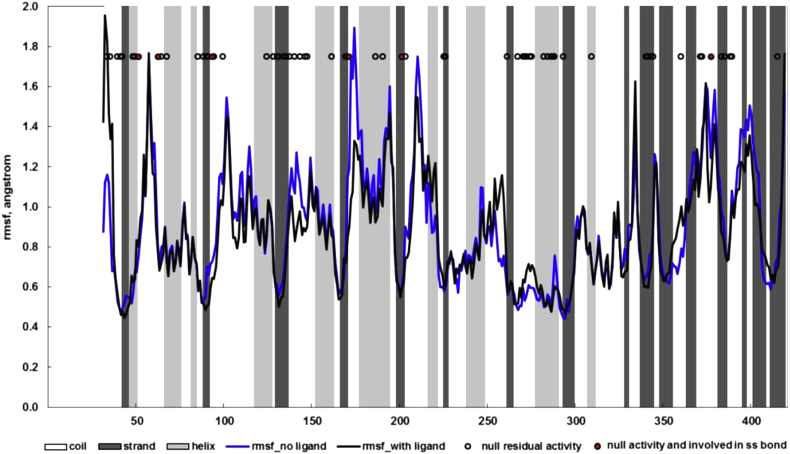
RMSF for the molecular dynamics run reported on the structure without the drug (blue line) or with the drug (black line). Circles represent sites where mutations are associated with null residual activity, the subset corresponding to cysteines involved in disulphide bonds is filled in red. Secondary structure is shown as background shading: white, coil; light gray, helix; dark gray, beta strand.

In Fig. 2 we show RMSF for the molecular dynamics run reported on the structure without the drug (blue line) or with the drug (black line).

There is little difference between the two. Notably the region where the highest difference is observed, spans aa 173–177. This observation is interesting because Asp 170 makes a salt bridge with the heterocyclic nitrogen in the drug molecule [30].

The beta strand of the TIM barrel at the tip of which are located the active site residues, forms the rigid core of the protein. The active site residues are rigid both in 3GXN and 3GXT. Circles represent sites where mutations are associated with null residual activity (first set). As can be observed, these sites are mostly found in regions corresponding to minima of flexibility or correspond to cysteines involved in disulphide bond formation (filled red circles in Fig. 2).

We correlated the residual AGAL activity measured in cells with the RMSF per residue obtained from running MD simulations on the substrate-bound 3GXT structure.

**Fig. 3:**
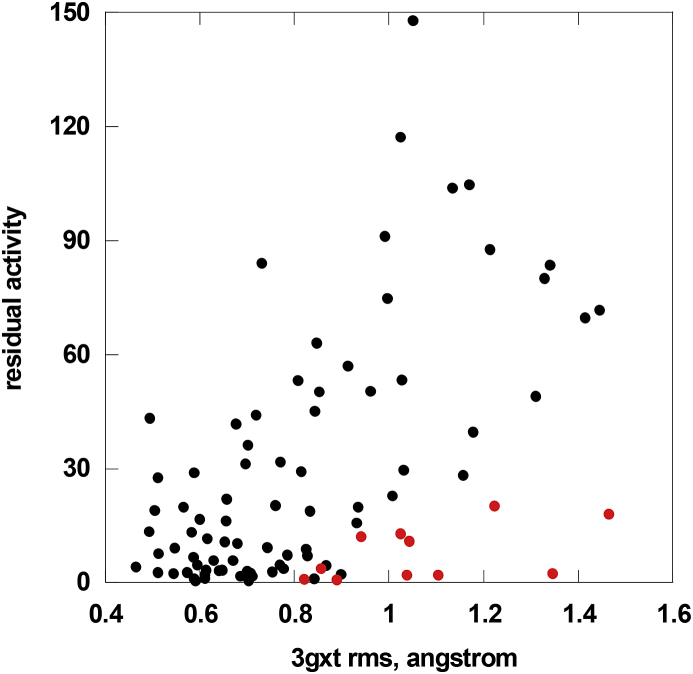
Correlation of residual AGAL activity measured in cells with the RMSF per residue obtained from running molecular dynamics on the 3GXT structure. Flexible residues that once mutated have activity lower than expected are shown (red circles).

As shown in Fig. 3 the correlation is medium (Pearson correlation coefficient R 0.50; p < 0.0001), but the trend is clear. When looking at this figure it should be born in mind that the experimental data come from several labs [22–28], who applied different methods and no attempt was made to exclude a priori those mutants that are known to have reduced k_cat_ or higher K_M_. Mutations occurring at flexible sites tend to be less severe. It is interesting to analyze the outliers, i.e. flexible residues that once mutated, have lower activity than expected (red in Fig. 3). Most of the outliers are residues for which abnormal kinetic parameters were determined (E59K or R112H) [29], residues lining the active site pocket (Y207) [31] or intrinsically flexible glycine residues (G35, G183, G258, G260, G261, G325, G360, G395) which can adopt unusual dihedral angles. Due to the correlation between flexibility and accessibility, it is not surprising that mutations occurring at exposed sites tend to retain residual activity. However it is not possible to find a molecular-level explanation to justify the numerous exceptions observed among buried as well as exposed sites (data not shown).

We gathered data on the activity of AGAL mutants in cells treated with DGJ [22–28]. Mutations occurring at the first group of sites, i.e. those with null residual activity, show little, if any, response to the drug, although exceptions are observed. For all mutations occurring at the second group of sites, i.e. those retaining residual activity, exposure to the drug is beneficial. However, the increase of activity is highly variable and does not correlate with RMSF (data not shown).

The approach presented here, validated through an extensive dataset for Fabry disease, is easy to generalize for any disease-related protein with available structural data. MD simulations as those used in the present study can nowadays almost be setup automatically and improved computing resources allow a swift execution of such runs. It remains to be assessed whether simpler and computationally even less demanding approaches may lead to correlations of comparable quality.

## 4 Concluding remarks

Fabry disease offers an exquisite example to prove the usefulness of chemico-physical methods for medicine. With regard to diagnosis, we correlated protein flexibility, predicted from MD simulations on the AGAL protein structure, with the residual activity of the pathological mutants measured in cells. Residual activity in turn correlates with the severity of the disease [20,21]. With our approach, we tried to overcome and enrich the binary “disease versus non-disease” classification of mutations. Our analysis reflects the spectrum of Fabry disease severity and helps identifying those patients who are eligible for pharmacological therapy. People affected by mutations that occur at very flexible sites should possess a relatively high residual activity and are likely to develop a mild and late form of the disease, people affected by mutations occurring at sites of intermediate flexibility should possess some residual activity and are likely to develop a mild form of the disease that is responsive to pharmacological chaperones [23]. People, in particular males, affected by mutations that occur at rigid sites, or affecting the active site or glycines with positive dihedral angles should have null residual activity and, consequently, severe and early onset Fabry disease. Nonetheless even in the less favorable case, some mutations might recover activity upon treatment with pharmacological chaperones [23–28]. The need of a graduated prediction of disease mutations will be more and more urgent when exome sequencing becomes largely employed. The case offered by Fabry disease with more than 400 missense mutations and large phenotypical differences will become common among genetic diseases. It will not be sufficient to classify a mutation as pathological one, but it will be necessary to predict whether the symptoms associated to a given genotype are severe, and which type of therapy is most appropriate.

## Competing interests

Maria Vittoria Cubellis was a consultant for Shire HGT in 2012.
